# Enhancing existing medical school curricula with an innovative healthcare disparities curriculum

**DOI:** 10.1186/s12909-021-03034-7

**Published:** 2021-12-11

**Authors:** Sean Treacy-Abarca, Marisela Aguilar, Stefanie D. Vassar, Estebes Hernandez, Neveen S. El-Farra, Arleen F. Brown

**Affiliations:** 1 UCLA David Geffen School of Medicine, Los Angeles, USA; 2 UCLA Fielding School of Public Health, Los Angeles, USA; 3 UCLA Division of General Internal Medicine & Health Services Research, Los Angeles, CA USA; 4 Olive View-UCLA Medical Center, Sylmar, CA USA; 5 Department of Medicine, David Geffen School of Medicine, University of California, Los Angeles, CA Los Angeles, USA; 6 UCLA Division General Internal Medicine and Health Services Research, 911 Broxton Plaza, Room 205, CA 90024 Los Angeles, USA

**Keywords:** Healthcare disparities, Medical education, Minority health

## Abstract

**Background:**

Effective healthcare disparities curricula seek to train physicians who are well equipped to address the health needs of an increasingly diverse society. Current literature on healthcare disparities curricula and implementation focuses on courses created independent of existing educational materials. Our aim was to develop and implement a novel resource-conserving healthcare disparities curriculum to enhance existing medical school lectures without the need for additional lectures.

**Methods:**

This non-randomized intervention was conducted at the University of California Los Angeles. The curriculum was offered to all first-year medical students in the class of 2021 (n=188). With institutional approval, a new healthcare disparities curriculum was created based on the Society of General Internal Medicine’s core learning objectives for effective healthcare disparities curricula (J General Internal Med 25:S160–163, 2010). Implementation of the curriculum made use of “teachable moments” within existing medical school lectures. Teachable moments were broad lecture topics identified by the research team as suitable for introducing relevant healthcare disparities content. The new lecture-enhancing healthcare disparities curriculum was delivered with the related lecture via integrated PDF documents uploaded to an online learning management system. Students were encouraged to complete pre- and post- course assessments to examine changes in disparities knowledge and self-rated confidence in addressing disparities. Matched χ2 tests were used for statistical analysis.

**Results:**

Participating students (n=92) completed both pre- and post-course assessments and were retrospectively stratified, based on self-reported use of the new lecture enhancing curriculum, into the “high utilizer” group (use of materials “sometimes” or “very often,” n=52) and the comparison “low utilizer” group (use of the materials “rarely” or “very rarely,” n=40). Students who self-identified as underrepresented racial and ethnic minorities in medicine were more likely to utilize the material (41% of the high utilizers vs. 17% of the low utilizer group, p<.01). Post-course knowledge assessment scores and self-reported confidence in addressing healthcare disparities improved only in the high utilizer group.

**Conclusions:**

Integrating new guideline based curricula content simultaneously into pre-existing lectures by identifying and harnessing teachable moments may be an effective and resource-conserving strategy for enhancing healthcare disparities education among first year medical students.

**Supplementary Information:**

The online version contains supplementary material available at 10.1186/s12909-021-03034-7.

## Introduction and background

Medical school curricula on the healthcare disparities that disproportionately affect racial and ethnic minority patients are critical for training well rounded, culturally sensitive physicians who are equipped to address the health needs of an increasingly diverse society. Current medical school curricula largely lack systematic teaching on evidenced based healthcare disparities topics. Early exposure to healthcare disparities topics for physicians in training is important for the goals of providing access to high quality care to all patient populations and ensuring that healthcare delivery is continuously improved. Although many have called for systematic introduction of healthcare disparities curricula in medical education, there remain limited data on successful strategies for achieving this goal [1–4]. To promote medical education reform to better address our society’s health needs, the Accreditation Council for Graduate Medical Education and the Liaison Committee on Medical Education have long advocated for effective healthcare disparities education to improve care for underserved patient populations [1, 2]. Healthcare disparity education has been described as suboptimal by both faculty and students despite available guidelines and the evidence of benefits for medical institutions that such curricula provide [3–5].

Effective healthcare disparity education also has positive effects for medical schools. Explicit attention to health disparities in the curriculum may improve recruitment of underrepresented in medicine minority (URM) students (those identifying racially or ethnically as African American and/or Black, Hispanic/Latino, Native American) [6]. Healthcare disparities curricula can promote a sense of inclusion for URM students in their learning environment, contributing to a diversified physician workforce that is better equipped to care for diverse patient populations [7–9]. Additionally, healthcare disparities education can more consistently portray minority populations who may not be represented among medical school faculty, staff, or students [10, 11]. Comprehensive education that includes minority health in a physician’s formative pre-clinical years improves patient outcomes [12–14]. However, vague or open-ended healthcare disparities curricula may impede non-URM students’ learning and comfort related to healthcare disparities [15]. Thus, both URM and non-URM students benefit from healthcare disparities curricula delivered with well-defined learning objectives.

The Society of General Internal Medicine (SGIM) Health Equity Commission, formerly the Disparities Task Force (DTF), proposed an approach for improving health disparities education in medicine by providing institutional stakeholders with well-defined learning objectives for effective healthcare disparities curricula [16]. These core objectives include:

1. Understand attitudes such as mistrust, subconscious bias and stereotyping that practitioners and/or patients may bring to the clinical encounter.
2. Attain knowledge of the existence and magnitude of health disparities, including the multi-factorial etiologies of and the multiple solutions required to eliminate them.
3. Acquire the skills to effectively communicate and negotiate across cultures, including trust-building and the use of key tools to improve cross-cultural communication [16].

Vela et al. used Health Equity Commission learning objectives to implement and evaluate a week-long course including lectures, small groups, clinic visits and poster presentations for incoming medical students [13, 17] This robust curriculum improved students’ knowledge and comfort with healthcare disparities. The study relied on trained, dedicated instructors, a workforce that may not be available at all institutions. In contrast, single standalone lectures and elective courses on healthcare disparities delivered alongside existing medical lectures have not been shown to be effective; instead, studies suggest that there may be benefit to diffusing these elements throughout medical school curricula [18–21]. No studies have examined whether the content of existing medical school lectures can provide “teachable moments,” i.e., the necessary context and opportunity to longitudinally integrate healthcare disparities teaching. Further, few studies have examined demographic differences in level of interest in disparities curriculum among medical students.

Broadly, our aim was to introduce a new implementation strategy to integrate guideline-based healthcare disparities content into an existing medical education curriculum. An important goal of this project was to address the societal need for physicians who are well equipped to address healthcare disparities in their direct service to patients and in their efforts to improve healthcare systems. To address this gap, we developed, implemented, and evaluated an innovative healthcare disparities curriculum and implementation strategy built upon SGIM Health Equity Commission learning objectives [7, 13]. Through this work, we sought to enhance existing lecture materials in a resource conserving and sustainable manner through simultaneous delivery of new content that capitalized on teachable moments. We sought to evaluate our curriculum’s effectiveness in teaching healthcare disparities without introducing more lecture time or requiring additional lecturers.

## Methods

### Setting and participants

All entering first-year medical students at the University of California, Los Angeles David Geffen School of Medicine (DGSOM) (n=188) in the class of 2021 were provided access to the new curriculum during the 8-week introductory course, “Block 1.” Use of the curriculum was encouraged, but course exams did not include this content, which allowed us to gauge organic interest in the material and characteristics of the students who participated.

### Program description

After an initial assessment, research team of students and institutional leaders, identified the need for a cost conserving, effective healthcare disparities curriculum. A wider group of institutional stake holders were briefed and approved the implementation of an institution-wide non-randomized intervention. Study research associates (second and third year medical students) were then recruited and trained to assist in the development of the new healthcare disparities curriculum. Training of research associates consisted of three in-person training sessions provided by institutional stakeholders to ensure an understanding of the SGIM Health Equity Commission’s learning objectives.

The trained research team then evaluated all lectures within the 8-week “Block 1” introductory course for the presence of teachable moments to integrate health disparities topics that were selected based on the SGIM Health Equity Commission’s learning objectives. Teachable moments were defined as opportunities within existing lectures where broad topics could be used to introduce specific healthcare disparities learning objectives. As an example, in a preexisting immunology lecture that addressed the biological mechanisms of asthma, the lecture was enhanced with content on racial and ethnic healthcare disparities in asthma prevalence, morbidity, mortality and environmental exposures. Topics were introduced using a template based on SGIM guidelines for healthcare disparities content. Research associates applied the template to the teachable moments identified for assigned topics then determined whether there were two or more available peer-reviewed research studies on the topic to support health equity enhancement of the curriculum.

Not all lectures had teachable moments. Within the 8-week course there were six weeks of lectures that were amendable to enhancement. Lecture-enhancing healthcare disparity teaching materials were developed for 26 lectures of 43 total lectures given during the six-week period. The amount of content provided based on the three SGIM learning objectives varied on a week to week basis, but 213 topics were introduced of which 15.5% related to objective 1, 54.5% related to objective 2, and 30% related to objective 3 (Table 1).

**Table 1:** Curriculum content stratified by week and core content

	Lectures	Lectures Enhanced	Topic Totals	Learning Objective 1	Learning Objective 2	Learning Objective 3
				Mistrust, bias and Stereotyping	Existence and Magnitude of Health Disparity	Communication Across Cultures
Week 2	10	6	31	7 (23%)	14 (45%)	10 (32%)
Week 3	9	2	19	4 (21%)	9 (47%)	6 (32%)
Week 4	10	7	73	7 (9.6%)	44 (60%)	22 (30%)
Week 5	6	4	37	3 (8%)	25 (66%)	9 (24%)
Week 6	8	7	53	12 (23%)	24 (45%)	17 (32%)
**Total**	43	26 (60%)	213	33 (15.5%)	116 (54.5%)	64 (30%)

The curriculum was made easily accessible through the online learning management system, Gryphon, which contained all medical school course materials. Block 1 course chairs announced the importance of the new lecture-enhancing healthcare disparities materials and how to access them. All students were encouraged to study the materials and participate in the curriculum evaluation. Students were informed that participation was voluntary. The new healthcare disparities content was provided to students on Gryphon as PDFs (Supplementary Fig. 1). No changes to students’ schedule or lectures were made. Individual involvement of lecturers in the curriculum varied, but all referenced the availability of the material.

### Research design

The study was granted a “Category 1” exemption for research on the effectiveness of or the comparison among instructional techniques by the UCLA Institutional Review Board. Major aims of our project were to examine the feasibility of utilizing teachable moments, understand the effectiveness of our implementation strategy in teaching healthcare disparities topics, and gauge student interested in the curricular content. We conducted a non-randomized intervention, comparing participants who reported use of the material to those who did not using a difference in differences approach. Given the exploratory nature of the project and the institutional stakeholders’ desire to ensure that all interested students had access to the disparities curriculum, we decided to forgo traditional randomization and evaluated the innovative curriculum and implementation strategy using a non-randomized study design.

All students in the DGSOM in the class of 2021 (n=188) were given access to the lecture-enhancing materials; a subset of students (n=92) completed pre-and post- course assessments (Supplementary File 1). We measured students knowledge of healthcare disparities with 16 true/false knowledge-based questions and examined their self-reported confidence levels in addressing healthcare disparities in clinical settings with seven Likert scale questions. These evaluation questionnaires were developed by the lead authors based on past literature using similar approaches. Such evaluation approaches are have been effective in assessing healthcare disparities curricula [13]. At the end of the 8-week course, students were asked the same questions, but randomly ordered. Students were tested only on topics and content taught in new healthcare disparities curriculum. Students self-reported their use of the new learning materials which was then used to accomplish our goal of understanding organic interest in learning about healthcare disparities and to create comparison groups.

Among the 92 students who completed the assessments, we compared those who reported use of the disparities material “sometimes” to “very often” on the post-course assessments (“utilized materials”) to the 40 students who reported that they used the lecture enhancing material “rarely” or “very rarely” (“did not utilize the material” n=40). The former were considered to have been fully exposed to the innovative curriculum and implementation strategy. The remaining students were considered the comparison group. The 96 students who did not were not included in analysis of the curriculum, and we do not know the extent to which they used the disparities curriculum material. McNemar’s matched χ2 test was utilized to compare performance on individual knowledge questions for the “utilized material” group versus the “did not utilize the material” group. We also compared aggregated scores for the two groups using two sample t-test. Statistical analysis utilized predetermined cut-offs for statistical relevance at p<.05. All analysis was conducted using STATA version 16.0 (StataCorp, College Station, Texas).

## Results

The participants consisted of 30% URM students and 70% non-URM or other race (Table 2). Students who self-identified as URM were more likely to have utilized the healthcare disparities material (41% of the high utilizer group compared to 17% of the low utilizer group, p<.01), while Asian students did not differ in utilization of the curriculum (40% versus 37%, p<.215), and White students were more likely to not use the material (46% versus 15%, p<.001) (Supplementary Table 1).

**Table 2:** Program evaluation for improvement in student knowledge and confidence post curriculum

Knowledge of Health Disparities	High Utilizers N = 52			Low Utilizers N = 40			High vs. Low Utilizers
Average percent correct answers (SD)	69 (15.6)	80 (13.8)	0.001	69 (12.1)	70.0 (14.7)	0.692	0.0013
**Abilities & Confidence Post- Curriculum**	**Poor/Fair %**	**Good** **%**	**Very Good/ Excellent %**	**Poor/Fair %**	**Good %**	**Very Good/ Excellent %**	**X**^**2**^ **P-Value**
Rate your present ability to describe some health disparities among Blacks/African Americans in the United States?	20	53	27	37	57	6	0.048
Rate your present confidence in addressing health disparities issues in a clinical setting?	22	65	13	51	40	9	0.021
Rate your present ability to describe some health disparities among Native American and Alaskan Native populations in the United States?	54	41	4	77	23	0	0.076
Rate your present ability to describe some health disparities among Hispanic/Latino populations in the United States?	22	61	17	37	57	6	0.142
Rate your present ability to describe the impact of socioeconomic status on disease outcomes?	2	57	41	6	71	23	0.182
Rate your present ability to describe impact of commercially obtained insurance and government health insurance on health outcomes?	22	72	7	31	69	0	0.220
Rate your present ability to describe major barriers and drivers of health disparity?	2	70	28	9	71	20	0.332
Composite of 16 True-False Questions	Pre Correct %	Post Correct %	*two sample * *t-test p-value*	Pre Correct %	Post Correct %	*two sample * *t-test p-value*	*two sample * *t-test p-value*

Individual performance on the 16-knowledge based true/false questions (Supplementary Table 2) and responses on confidence based Likert questions was used to generate composite scores for high compared to low utilizers (Table 2). No statistically significant differences were found between the two groups in the pre-curriculum knowledge. The average composite post-curriculum knowledge score for the high utilizer group was 79.9%, compared to 70% in the low utilizer group (p<.001) (Table 2). Composite post-course knowledge scores for the high utilizer group improved by 10.8% from a baseline of 69.1%. Pre-course knowledge scores for the comparison group did not improve. The high utilizer group had higher self-reported confidence (“good” or “very good/excellent”) in addressing healthcare disparities issues in a clinical setting: 78%, compared to 49% for the comparison group, p<.02. Almost all respondents (96%) described the health disparities curriculum as a valuable learning resource. No data were available for the students who chose not to complete the assessments (n=96).

## Discussion

We present an innovative healthcare disparities curriculum that was implemented in a resource conserving manner by utilizing “teachable moments” to integrate a healthcare disparities curriculum into pre-existing medical school lectures. We observed improved knowledge of healthcare disparities topics and enhanced self-reported confidence in addressing these topics in a clinical setting. Both findings suggest that this approach may be an important tool for medical institutions interested in advancing equitable healthcare by promoting a heightened understanding of healthcare disparities among trainees.

Capitalizing on teachable moments is a viable resource-conserving strategy to implement guideline-based healthcare disparities curricula like ours. A key to harnessing teachable moments was use of an established guideline, a team with knowledge of healthcare disparities, and administrative stakeholders who facilitated the project. These features also are important components to generalize this work to other institutions in an efficient and sustainable manner.

An effective healthcare disparities curriculum during a physician’s formative pre-clinical years has the potential to contribute to the advancement of equitable healthcare for underserved populations. The evidence-based health disparities content in our intervention exposes physicians in training—with varying baseline knowledge and interest of healthcare disparities—to information that prepares them to care for patients from underserved socioeconomic and racial and ethnic backgrounds. In our study, students’ knowledge of healthcare disparities improved significantly as did their confidence in addressing these topics in a clinical setting. Incorporating this information into the curriculum is one step toward equipping physicians with the tools to address healthcare disparities and to contribute to solutions for the care of vulnerable populations.

URM students were more likely to utilize the learning materials than their non-URM counterparts. Understudied areas of medical education include the effects of healthcare disparities curricula on both the short and long-term success of URM students. Robust healthcare disparities curricula may facilitate recruitment of URM applicants to medical programs [7]. Further work should examine how healthcare disparities teaching affects URM student success in medical school and their long term outcomes. Although non-URM students were less likely to utilize the material, this finding provides an opportunity for future studies to examine strategies to increase self-motivated learning about healthcare disparities among non-URM students, who represent the majority of medical students nationwide. We hope findings such as ours motivate institutional stakeholders to create learning environments that ensure students from varying demographic backgrounds are well versed in the existence and importance of addressing healthcare disparities.

At the level of institutional stakeholders, our study builds upon existing approaches that have focused on the creation of standalone lectures and courses rather than enhancing existing medical school lectures. One successful example of a standalone course, implemented by *Vela et al.*, delivered an immersive healthcare disparities curriculum prior to first year course work [13]. Similar to our curriculum this course was built upon SGIM Health Equity Commission learning objectives. ^8^ However, in contrast we improved students’ knowledge of healthcare disparities and their confidence in addressing healthcare disparities without the creation of a standalone course. These analyses have some limitations. Our study intentionally used a convenience sample, which allowed us to study self-motivated learning for healthcare disparities across a diverse cohort of students. A randomized controlled trial can provide additional insights into the impact of the curricular enhancement and teachable moment approach. An alternate strategy is to promote participation in voluntary healthcare disparities curricula across all members of the student body. For example, providing additional benefits to students who voluntarily participate in this program may increase utilization of these enhanced curricula. This is a single center study, which limits external validity; however, our student cohort included substantial numbers of URM in medicine, who are an understudied population in medical education literature. As this project was an initial pilot study of the curriculum and implementation strategy, we made use true-false tests and simple metrics to assess gains in knowledge and confidence. Future work should incorporate more complex strategies, such as case-based evaluation. Continued evaluation during students’ clinical years is also important to study extinguishment/sustainment of the intervention effect, and longer term association of the curriculum with student knowledge, confidence, and attitudes. Finally, additional work should also be devoted to achieving consensus on core competencies that all medical students need to address healthcare disparities in their careers.

## Conclusions

By harnessing teachable moments and delivering content that enhances healthcare disparities curriculum concurrently with traditional lectures through an online learning management system, we were able to increase knowledge about healthcare inequities and self-rated confidence in addressing disparities during the 8-week course. Integrating these resource conserving approaches into existing online learning management systems is a viable strategy for many medical schools. The educational resources we developed have been made available to other programs via an open access online repository that will allow programs to tailor the curricula and strategies to their programs.

## Supplementary Information

**Additional file 1.**

**Additional file 2.**

**Additional file 3.**

**Additional file 4.** Health Disparities Assessment.

## Data Availability

The datasets used and/or analysed during the current study are available from the corresponding author on reasonable request.

## Abbreviations

DGSOM: David Geffen School of Medicine
DTF: Disparities Task Force
SGIM: Society of General Internal Medicine
URM: Underrepresented in Medicine Minority
