# Supplementary material for: Enhancing existing medical school curricula with an innovative healthcare disparities curriculum
Source: BMC Med Educ. 2021 Dec 11;21:613. doi: 10.1186/s12909-021-03034-7 (PMC8666085; doi:10.1186/s12909-021-03034-7)
Supplement: Supplementary file 1 — Additional file 1. [file 12909_2021_3034_MOESM1_ESM.docx]

Enhancing Existing Medical School Curricula with an Innovative Healthcare Disparities Curriculum

Supplementary File 1: Health Disparities Assessment

Q1 Black/African American pregnancies face the highest rate of neural tube defects when compared to their counterparts.

- True (1)
- False (2)

Q2 Blacks/African Americans and Hispanics/Latinos are overrepresented in Phase 1 clinical trials.

- True (1)
- False (2)

Q3 Due to increased awareness, Black/African American men who have sex with men (MSM) and White MSM are equally likely to report taking antiretroviral medications.

- True (1)
- False (2)

Q4 Medicare coverage does not ensure access to asthma related specialists.

- True (1)
- False (2)

Q5 Lupus incidence is highest among Hispanic women.

- True (1)
- False (2)

Q6 Medicaid provides the same health outcomes as commercially derived insurance for patients with severe illness and demonstrates the need for expansion of public health systems.

- True (1)
- False (2)

Q7 Patient satisfaction is lower if their provider is of different ethnic or racial background.

- True (1)
- False (2)

Q8 Cancer incidence but not cancer mortality rates exhibit differences across race and ethnicity.

- True (1)
- False (2)

Q9 Pediatric asthma patients of all races and ethnicities are equally likely to utilize emergency room departments for asthma exacerbations.

- True (1)
- False (2)

Q10 Although there is a greater incidence of diabetes among Black/African American and Latino/Hispanic populations, there are no differences in morbidity when compared to Whites.

- True (1)
- False (2)

Q11 The purpose of the Tuskegee Study was to provide free medical care to Blacks/African Americans suffering from syphilis in order to reduce health disparity.

- True (1)
- False (2)

Q12 Lack of insurance, but not negative perceptions of healthcare staff are prevalent barriers to prenatal care for Black/African American and Latina/Hispanic women.

- True (1)
- False (2)

Q13 All minority populations are equally at a greater risk for Hepatitis B infection and related morbidities.

- True (1)
- False (2)

Q14 Minority patients with diabetes face greater incidence of diabetic renal disease when compared to Whites.

- True (1)
- False (2)

Q15 Access to cancer related surgical procedures tends to be based on socioeconomic status.

- True (1)
- False (2)

Q16 The 1996 Folate Fortification helped to eliminate folate related health disparity.

- True (1)
- False (2)

Q17 Rate your present ability to describe some health disparities among Blacks/African Americans in the United States?

- Poor/Fair (1)
- Good (2)
- Very Good/Excellent (3)

Q18 Rate your present confidence in addressing health disparities issues in a clinical setting?

- Poor/Fair (1)
- Good (2)
- Very Good/Excellent (3)

Q19 Rate your present ability to describe some health disparities among Native American and Alaskan Native populations in the United States?

- Poor/Fair (1)
- Good (2)
- Very Good/Excellent (3)

Q20 Rate your present ability to describe some health disparities among Hispanic/Latino populations in the United States?

- Poor/Fair (1)
- Good (2)
- Very Good/Excellent (3)

Q22 Rate your present ability to describe the impact of socioeconomic status on disease outcomes?

- Poor/Fair (1)
- Good (2)
- Very Good/Excellent (3)

Q24 Rate your present ability to describe impact of commercially obtained insurance and government health insurance on health outcomes?

- Poor/Fair (1)
- Good (2)
- Very Good/Excellent (3)

Q25 Rate your present ability to describe major barriers and drivers of health disparity?

- Poor/Fair (1)
- Good (2)
- Very Good/Excellent (3)
