# Supplementary material for: Enhancing existing medical school curricula with an innovative healthcare disparities curriculum
Source: BMC Med Educ. 2021 Dec 11;21:613. doi: 10.1186/s12909-021-03034-7 (PMC8666085; doi:10.1186/s12909-021-03034-7)
Supplement: Supplementary file 2 — Additional file 2. [file 12909_2021_3034_MOESM2_ESM.docx]

| **Supplementary Table 1: Comparison of Pre- and Post-Course Knowledge of Health Disparities High Utilizer versus Low Utilizer Learners** | | | | | | | |
| --- | --- | --- | --- | --- | --- | --- | --- |
|  | **High Utilizers**  **N = 52** | | | **Low Utilizers**  **N = 40** | | | **Utilized vs. Not Utilized Post** |
|  | **Pre**  **% Correct** | **Post**  **% Correct** | **Matched**  **McNemar’s X^2^ Test P-Value** | **Pre**  **% Correct** | **Post**  **% Correct** | **Matched**  **McNemar’s X^2^ Test P-Value** | **X^2^ P-Value** |
| **1.** Black/African American pregnancies face the highest rate of neural tube defects when compared to their counterparts. | 12 | 40 | 0.003 | 13 | 20 | 0.508 | 0.037 |
| **2.** Blacks/African Americans and Hispanics/Latinos are overrepresented in Phase 1 clinical trials. | 37 | 62 | 0.019 | 30 | 30 | 0.999 | 0.003 |
| **3.** Lupus incidence is highest among Hispanic women. | 38 | 48 | 0.359 | 28 | 28 | 0.999 | 0.045 |
| **4.** Due to increased awareness, Black/African American men who have sex with men (MSM) and White MSM are equally likely to report taking antiretroviral medications. | 85 | 94 | 0.227 | 88 | 85 | 0.999 | 0.140 |
| **5.** Medicare coverage does not ensure access to asthma related specialists. | 67 | 85 | 0.049 | 70 | 70 | 0.999 | 0.092 |
| **6.** Medicaid provides the same health outcomes as commercially derived insurance for patients with severe illness and demonstrates the need for expansion of public health systems. | 56 | 69 | 0.210 | 55 | 58 | 0.999 | 0.245 |
| **7.**Patient satisfaction is lower if their provider is of different ethnic or racial background. | 77 | 85 | 0.424 | 73 | 75 | 0.999 | 0.249 |
| **8.** Cancer incidence but not cancer mortality rates exhibit differences across race and ethnicity. | 73 | 87 | 0.144 | 60 | 70 | 0.424 | 0.052 |
| **9.** Pediatric asthma patients of all races and ethnicities are equally likely to utilize emergency room departments for asthma exacerbations. | 77 | 94 | 0.012 | 90 | 88 | 0.999 | 0.256 |
| **10.** Although there is a greater incidence of diabetes among Black/African American and Latino/Hispanic populations, there are no differences in morbidity when compared to Whites. | 96 | 96 | 0.999 | 88 | 93 | 0.727 | 0.443 |
| **11.** The purpose of the Tuskegee Study was to provide free medical care to Blacks/African Americans suffering from syphilis in order to reduce health disparity. | 81 | 96 | 0.008 | 90 | 93 | 0.999 | 0.443 |
| **12.** Lack of insurance, but not negative perceptions of healthcare staff are prevalent barriers to prenatal care for Black/African American and Latina/Hispanic women. | 81 | 85 | 0.480 | 88 | 88 | 0.999 | 0.694 |
| **13.** All minority populations are equally at a greater risk for Hepatitis B infection and related morbidities. | 90 | 83 | 0.344 | 90 | 83 | 0.453 | 0.981 |
| **14.** Minority patients with diabetes face greater incidence of diabetic renal disease when compared to Whites. | 100 | 98 | 0.999 | 100 | 98 | 0.999 | 0.851 |
| **15.** Access to cancer related surgical procedures tends to be based on socioeconomic status. | 98 | 96 | 0.999 | 100 | 98 | 0.999 | 0.719 |
| **16.** The 1996 Folate Fortification helped to eliminate folate related health disparity. | 38 | 62 | 0.023 | 40 | 48 | 0.648 | 0.179 |
