# Supplementary material for: Enhancing existing medical school curricula with an innovative healthcare disparities curriculum
Source: BMC Med Educ. 2021 Dec 11;21:613. doi: 10.1186/s12909-021-03034-7 (PMC8666085; doi:10.1186/s12909-021-03034-7)
Supplement: Supplementary file 3 — Additional file 3. [file 12909_2021_3034_MOESM3_ESM.docx]

| **Supplementary Table 2: Comparison of Pre-Course Knowledge of Health Disparities Between High Utilizer Versus Low Utilizer Learners** | | | |
| --- | --- | --- | --- |
|  | **High Utilizers**  **N = 52**  **Pre-Course % Correct** | **Low Utilizers**  **N = 40**  **Pre-Course % Correct** | **Chi-2 Test P-Value** |
| **1.** Black/African American pregnancies face the highest rate of neural tube defects when compared to their counterparts. | 12 | 13 | 0.888 |
| **2.** Blacks/African Americans and Hispanics/Latinos are overrepresented in Phase 1 clinical trials. | 37 | 30 | 0.511 |
| **3.** Lupus incidence is highest among Hispanic women. | 38 | 28 | 0.270 |
| **4.** Due to increased awareness, Black/African American men who have sex with men (MSM) and White MSM are equally likely to report taking antiretroviral medications. | 85 | 88 | 0.694 |
| **5.** Medicare coverage does not ensure access to asthma related specialists. | 67 | 70 | 0.783 |
| **6.** Medicaid provides the same health outcomes as commercially derived insurance for patients with severe illness and demonstrates the need for expansion of public health systems. | 56 | 55 | 0.941 |
| **7.**Patient satisfaction is lower if their provider is of different ethnic or racial background. | 77 | 73 | 0.627 |
| **8.** Cancer incidence but not cancer mortality rates exhibit differences across race and ethnicity. | 73 | 60 | 0.185 |
| **9.** Pediatric asthma patients of all races and ethnicities are equally likely to utilize emergency room departments for asthma exacerbations. | 77 | 90 | 0.101 |
| **10.** Although there is a greater incidence of diabetes among Black/African American and Latino/Hispanic populations, there are no differences in morbidity when compared to Whites. | 96 | 88 | 0.121 |
| **11.** The purpose of the Tuskegee Study was to provide free medical care to Blacks/African Americans suffering from syphilis in order to reduce health disparity. | 81 | 90 | 0.222 |
| **12.** Lack of insurance, but not negative perceptions of healthcare staff are prevalent barriers to prenatal care for Black/African American and Latina/Hispanic women. | 81 | 88 | 0.386 |
| **13.** All minority populations are equally at a greater risk for Hepatitis B infection and related morbidities. | 90 | 90 | 0.951 |
| **14.** Minority patients with diabetes face greater incidence of diabetic renal disease when compared to Whites. | 100 | 100 | N/A |
| **15.** Access to cancer related surgical procedures tends to be based on socioeconomic status. | 98 | 100 | 0.378 |
| **16.** The 1996 Folate Fortification helped to eliminate folate related health disparity. | 38 | 40 | 0.881 |
| **Composite of 16 True-False Questions** |  |  | *two sample  t-test p-value* |
| **Average percent correct answers (SD)** | 69.1 (15.6) | 68.8 (12.1) | **.904** |
