# Supplementary material for: Enhancing existing medical school curricula with an innovative healthcare disparities curriculum
Source: BMC Med Educ. 2021 Dec 11;21:613. doi: 10.1186/s12909-021-03034-7 (PMC8666085; doi:10.1186/s12909-021-03034-7)
Supplement: Supplementary file 4 — Additional file 4. Health Disparities Assessment. [file 12909_2021_3034_MOESM4_ESM.docx]

| **Supplementary Figure 1:** Example lecture enhancing healthcare disparities material |
| --- |
|  |
| a-c) Example of lecture enhancing healthcare disparities curriculum content provided to students on an online learning management system where traditional lecture material was provided. This PDF was downloaded at the same time as all other lecture materials (lecture slides, lecture handouts, article readings, lecture video recordings). |
